# A Proposal for the Interpretation of Serum IGF-I Concentration as Part of Laboratory Screening in Children with Growth Failure

**DOI:** 10.4274/jcrpe.galenos.2019.2019.0176

**Published:** 2020-06-03

**Authors:** Jan M. Wit, Martin Bidlingmaier, Christiaan de Bruin, Wilma Oostdijk

**Affiliations:** 1 Leiden University Medical Center, Department of Paediatrics, Leiden, The Netherlands; 2 Klinikum der Universität München, Medizinische Klinik und Poliklinik IV, Endocrine Research Laboratories, Munich, Germany

**Keywords:** Short stature, growth disorders, IGF-I, IGFBP-3

## Abstract

The serum insulin-like growth factor-I (IGF-I) concentration is commonly used as a screening tool for growth hormone deficiency (GHD), but there is no consensus on the cut-off limit of IGF-I standard deviation score (SDS) to perform GH stimulation tests for confirmation or exclusion of GHD. We argue that the cut-off limit is dependent on the clinical pre-test likelihood of GHD and propose a diagnostic strategy in which the cut-off limit varies between zero to -2 SDS.

## Introduction

In guidelines on the diagnostic approach to children referred to a paediatrician or paediatric endocrinologist for short stature and/or growth faltering (from now on referred to as “growth failure”, abbreviated as GF), including the consensus paper on Idiopathic short stature (ISS) (1) and the recent Dutch guideline (2), it is advised to perform laboratory screening for potential subclinical pathological causes, including serum insulin-like growth factor-I (IGF-I) [with or without serum IGF-binding protein-3 (IGFBP-3)], in order to screen for growth hormone deficiency (GHD). During the pre-final phase of the Dutch guideline, when authorization was sought from representatives of the Paediatric Association of the Netherlands and other specialist societies, the working group was asked to add specific instructions for the general paediatrician at which cut-off point of IGF-I a GH stimulation test should be performed to confirm or exclude GHD. Since a literature search did not provide a clear answer to this question, we performed a stepwise analysis of the decision procedure, taking established clinical epidemiological techniques into consideration. This led to several subsequent versions of an addendum to the Dutch Guideline on Triage and Diagnosis of Growth disorders in children (2), which were reviewed by members of the Section of Paediatric Endocrinology and the Growth Hormone Advisory Group of the Paediatric Association of the Netherlands and discussed at several meetings. In the present commentary we present an English adaptation of the addendum to the official guideline that will be published in Dutch on the internet.

## Definition, Subcategories and Varying Levels of Uncertainty of GHD

Impaired secretion of GH in children is causally related to impaired growth, anthropometric characteristics (e.g., normal proportions, relatively large head) and changes of body composition (e.g., sarcopenia, excess of body fat and low bone mineral density) as well as functional abnormalities (e.g., hypoglycaemia) (3,4). However, for practical and financial reasons it is troublesome to measure the GH secretion over 24 hours in individual patients, so that as a proxy indicator of spontaneous GH secretion the GH response to a stimulus is commonly used. The various problems of such stimulation tests are well known (5) and also low serum levels of GH-dependent proteins (IGF-I and IGFBP-3) cannot be considered gold standards.

GHD can be subcategorized into acquired and non-acquired (congenital) forms (4). The acquired form is usually caused by space-occupying processes in the brain, such as tumours (e.g., craniopharyngioma), but can also be the consequence of brain trauma, infections, irradiation, histiocytosis or vascular anomalies. The congenital form can be subdivided into three subgroups (6). The first is an (almost) certain form where the etiology is known, for example if GHD is associated with cerebral or facial malformations, anatomic pituitary or hypothalamic abnormalities detected with magnetic resonance imaging (MRI), other pituitary hormone deficiencies, or established genetic causes of GHD, such as mutations of *GH1*, *GHRHR* or *GHSR* in isolated GHD (IGHD) and of multiple transcription factors in multiple pituitary hormone deficiency. In line with Ranke et al (6) this etiologic subgroup is abbreviated as cGHD. The second and most frequent subgroup is idiopathic IGHD (IIGHD). The third subgroup is growth hormone neurosecretory dysfunction (NSD), characterised by clinical features (including a “typical” growth curve) and low serum IGF-I which lead to suspicion of GHD, in combination with a normal GH peak during a stimulation test. In the original papers it was shown that such children had a low spontaneous GH secretion over 24 hours in contrast to a normal GH peak in response to a GH stimulation test (7,8,9). Due to the practical and financial hurdles of performing 24-hour GH profiles, this diagnosis has also been assumed without proof of a decreased spontaneous GH secretion (6).

The diagnostic process of acquired GHD and cGHD is straightforward, although sometimes with considerable diagnostic delay, but this does not apply to IIGHD and NSD. Retesting of the hypothalamic-pituitary-GH/IGF-I axis in puberty and after reaching adult height of patients with IIGHD leads in most cases to normal results regarding the GH peak in a GH stimulation test (10,11,12). This phenomenon is usually interpreted as an initial false-positive result of GH testing, although a transient form of GHD cannot be excluded. The diagnosis of NSD has been controversial from the beginning, although an adequate growth response to GH treatment of children diagnosed as NSD has been documented (7,8,9).

## Diagnostic Approach with Respect to GHD in Children Referred for Growth Failure

There is no doubt that the diagnostic process for children referred for GF should be aimed at detecting and treating all children with acquired GHD or cGHD as soon as possible. In the remaining children (IIGHD and NSD), which constitute the majority of cases (6), the clinician has to face the challenge to distinguish as best as possible IIGHD or NSD from other causes of GF or short stature of unknown origin (ISS). In children it is particularly difficult to distinguish IIGHD from the non-familial form of ISS with maturational delay (1,3,4). Many prepubertal short children who later present with delayed puberty, have a prepubertal growth pattern characterised by a low height velocity [decreasing height standard deviation score (SDS)] (13,14). However, the diagnosis of “Constitutional Delay of Growth and Puberty” can only be diagnosed with certainty if the onset of puberty (Tanner genital or breast stage 2) is indeed delayed (boys >14 years, girls >13 years).

For an estimate of the probability of GHD in a child with GF (in comparison to another or unknown cause), the clinician needs to take the following steps:

1. Be aware of the general prevalence of GHD in children referred for GF in the specific clinic;

2. Estimate the *individual pre-test likelihood of GHD* in the patient, based on weighing the diagnostic clues from the medical history (including family history), physical examination, growth curve, laboratory screening and skeletal maturation;

3. Perform *laboratory screening* using serum IGF-I and (in young children) IGFBP-3, and interpret the result taking into consideration age, sex, pubertal status and body mass index;

4. Calculate the *post-test likelihood of GHD* based on serum IGF-I (with or without IGFBP-3) and individual pre-test likelihood;

5. If the post-test likelihood is considered sufficiently high, perform GH-provocation tests (GH-stimulation tests);

6. If the results of the GH stimulation test are compatible with GHD, perform an *MRI of the hypothalamus-pituitary area* (and in specific cases genetic testing can also be considered); 

7. If the GH peak is normal in the setting of a severely decreased serum IGF-I, consider performing an IGF-I generation test (to differentiate between rare syndromes with either normal or decreased GH sensitivity) and/or genetic tests, preferably after consultation with a paediatric endocrinologist and/or clinical geneticist knowledgeable in genetic causes of GF. 

In this Critical Review we shall discuss the first four steps. For the remaining three steps we refer to previous documents, references 15,16,17,18.

## Prevalence of GHD and Its Subcategories in Children Referred for Growth Failure

The prevalence of GHD in the general population of children is uncertain. Estimates vary from 1:3,400 to 1:30,000 (19,20,21,22,23). If one estimates a prevalence of 1:5000, and assumes that almost all cases present with a height below -2 SDS (2.3rd percentile), the prevalence of GHD in short children in the population would be approximately 1%. The reported prevalence of GHD in children referred for GF to a paediatric (endocrine) clinic also varies. In Dutch studies the prevalence was between 0.5-3% (24,25,26) and in other studies prevalences between 0 and 23% were reported (27,28,29,30,31,32,33,34). For this analysis we estimated the general prevalence of GHD in children referred for GF at 2%, in line with a recent publication (35), but this percentage may differ between a general and academic paediatric clinic. Furthermore, the variation in diagnostic approaches, for example whether sex hormone priming is used when a GH stimulation test is performed in the pre- to early-pubertal age range (36), can also be expected to have a significant impact on the percentage of GHD diagnosed in referred children.

We have not found data on the proportion of acquired and congenital GHD, but based on our clinical experience, acquired GHD is rare. Within the group of congenital GHD an indication of the relative proportions can be obtained from the report on 50 years’ experience in Tübingen: out of 636 patients, 122 had cGHD (19.2%), 455 IIGHD (71.7%) and 58 (9.1%) NSD (6).

## Estimate of the Individual Pre-test Likelihood of GHD in a Patient Referred for Growth Failure

A full clinical assessment of the child will lead to an estimate of the individual clinical pre-test likelihood of GHD, based on an inventory of all relevant clinical features. These comprise diagnostic clues from the medical history, physical examination, growth curve, general laboratory screening and skeletal age. Although objective data are lacking, we believe that the relative weight of these diagnostic clues is different. Table 1 shows the diagnostic clues and our personal opinion on their relative importance. Future prospective studies are needed to collect observational data on the relative weight of the various diagnostic clues. Based on the balance of positive and negative diagnostic clues, the clinician can estimate the clinical pre-test likelihood of GHD in an individual patient. Thereafter, the result of serum IGF-I (and IGFBP-3) can be interpreted against the background of the pre-test likelihood of GHD.

The estimate of the pre-test likelihood of GHD is obviously a continuum and subjective, since objective data are lacking for the proportional weighting of diagnostic clues. For practical purposes, we propose five categories of pre-test likelihood:

1. High pre-test likelihood (≥≈50%), if there are several positive clues and no negative clues.

2. Moderate pre-test likelihood (≈20%), if there are some positive clues for GHD, and no negative clues.

3. Rather low pre-test likelihood (≈10%), if there are few positive clues for GHD and no negative clues.

4. Low pre-test likelihood (≈5%), if there are few positive clues and at least one negative clue for GHD.

5. Very low pre-test likelihood (≤≈2%), if there are no positive clues and/or ≥1 negative clues for GHD; this is roughly equal to (or less than) the estimated prevalence in children referred for GF.

In case of a high pre-test likelihood of GHD the clinician will directly perform an MRI of the pituitary/hypothalamic area, particularly in neonates or if there is clinical suspicion of acquired GHD. In neonates with clinical and biochemical features of hypothalamic/pituitary deficiency an abnormal brain MRI in combination with a random serum GH concentration <7 µg/L in the first week of life has been proposed as sufficient to make the diagnosis of GHD (37). A low serum IGF-I supports the diagnosis, but its specificity at that age is low because the lower limit of the reference range is then below the sensitivity of the assay. A low IGFBP-3 (<-2.0 SDS) is also supportive, and in general its specificity appears to be higher than that of IGF-I (38,39,40).

In addition, when an acquired form of GHD is suspected, the clinician will promptly perform a brain MRI, as well as further diagnostic investigations into hypothalamic/pituitary function. Such investigations will usually be repeated following neurosurgical or oncological treatment. During follow-up of neuro-oncological patients with an increased risk of developing GHD, a recent international guideline advised to perform GH stimulation tests when there is clinical suspicion of GHD, irrespective of the result of serum IGF-I, because in such patients a low serum IGF-I appears to have a low sensitivity (41). It has been suggested that if multiple pituitary axes are affected, a GH stimulation test can be omitted because of the high pre-test likelihood of GHD (41). In patients with possible IIGHD, a brain MRI is only performed after GHD has been confirmed by GH stimulation tests.

## Laboratory Screening Using Serum IGF-I and Interpreting the Result Adjusting for Age, Sex, Pubertal Status and Body Mass Index

The serum concentration of IGF-I is not only influenced by GH secretion, but also by biological factors, such as nutritional status, age, sex, and pubertal stage, as well as analytical factors. This implies that if the serum IGF-I concentration is measured in the context of laboratory screening as an indicator of GHD in children with GF, the result should first be adjusted for confounding factors.

In most countries different commercial IGF-I assays are used with a considerable inter-assay variation. In our country, most of the inter-assay variation of 5-20% is compensated for because of a national harmonization program. For each assay there is also some intra-assay variation [e.g., a coefficient of variation of 6-8% in the assay used in Tübingen (6) and a total coefficient of variation 3.4-8.7% in the iSYS assay (42)], so that repetition of an IGF-I determination should be considered, particularly if there is a discrepancy between the clinical features and the laboratory results. Given the inter-assay variation, it is advisable to use the same assay during follow-up of an individual patient.

For a proper interpretation of the serum IGF-I concentration, the first step is to express IGF-I as SDS for age and sex. Obviously, for this purpose adequate reference data from a large population of healthy children are needed. Given the differences between assays, reference data should be gathered for each assay separately.

It is generally assumed that in prepubertal children in the age range where puberty usually has not yet started IGF-I SDS can be calculated for age and sex. However, the maximum age for this approach is arbitrary. One could argue that the 10th percentile of the age at start of puberty in the population would be a rational choice (9 years in girls and 10 years in boys in our country), but other investigators suggested a cut-off of 8 and 9 years, respectively (35).

Regarding adjustment for pubertal status in older children, the situation is more complex, and a further adjustment step has to be performed. For this purpose, there are essentially two possible approaches. For a few assays reference data have been reported on IGF-I per pubertal stage [for example (42,43,44)], but the assays used in the two oldest reports were calibrated to old standards. IGF-I percentiles according to pubertal (Tanner) stage for the IDS iSYS assay are shown in Table 2 (42).

If an assay is used for which such reference data are unavailable, one can calculate SDS for skeletal age (35), because a (relatively) delayed puberty is usually associated with a (relatively) delayed skeletal age. However, its predicted power is reportedly slightly lower than that of the SDS for pubertal stage (35).

Since the expression of IGF-I as an SDS is more informative than a percentile position, we calculated mean, SD and cut-off limits for various SDS for children with different Tanner stages based on the raw data that were used for the construction of reference data for the IDS iSYS assay (Table 3) (42).

A further complication when assessing serum IGF-I occurs if BMI SDS is low or high (45). In a child with a low BMI SDS, another sign of undernutrition, or a recent disease associated with decreased appetite, IGF-I is relatively low. This would imply that in such children a low serum IGF-I has a relatively low predictive value. If feasible, one may wish to first improve the nutritional condition before repeating further IGF-I measurements. In a child or adult with a high BMI SDS, GH secretion is usually low in contrast to a serum IGF-I in the upper half of the reference range. In overweight children, one could therefore expect that the optimal cut-off point of IGF-I for the decision to perform a GH stimulation test may be higher than in children with a normal BMI SDS. Due to the lack of observational data, a formal adjustment of the IGF-I result for BMI is not possible, so that the clinician can only use his/her subjective clinical judgement.

When a low serum IGF-I is found in a child with a low clinical pre-test likelihood we suggest to repeat serum IGF-I, preferably in combination with a determination of serum IGFBP-3, before one decides to perform a GH stimulation test. If IGFBP-3 SDS is remarkably lower than IGF-I SDS, one should consider the possibility of a homozygous mutation of *IGFALS* (46).

## Calculation of the Post-test Likelihood of GHD Based on Serum IGF-I (with or without IGFBP-3) and Individual Pre-test Likelihood of GHD

In a previous study by our group (47), various parameters for quantifying the diagnostic value of serum IGF-I and IGFBP-3 and other markers were investigated in a Dutch cohort of children from four years of age with GHD or ISS. The optimal cut-off point for IGF-I for the diagnosis of GHD was -0.83 SDS [with an area under the curve (AUC) of 0.80 in the ROC analysis], but specificity was low at that point (47%). The optimal cut-off point for IGFBP-3 was -0.47 SDS (AUC 0.69) with a specificity of 22%. In the same publication data were shown on the frequencies of a serum IGF-I or IGFBP-3 SDS below <-2, <-1 and <0 in children with either GHD or ISS, as well as the positive likelihood ratio (LR+) and negative likelihood ratio (LR-) (Table 4).

For IGF-I, the sensitivity (65%) and specificity (78%) of a cut-off at -2 SDS in the Dutch study (47) were similar to those reported in other studies, as summarized in a meta-analysis (39), where average sensitivity and specificity of IGF-I were 0.66 and 0.69, with positive and negative LRs similar to those in the Dutch study. Also for IGFBP-3 similar findings were reported in the Dutch study (sensitivity 53%, specificity 81%) as in the later meta-analysis (50% and 79%). It is noteworthy that a low IGFBP-3 is less sensitive but more specific than a low IGF-I.

In the literature there is no consensus whether IGFBP-3 should be added to an IGF-I measurement in the screening phase (48). In light of the higher sensitivity of IGF-I (38,39), and in order to reduce expenses, in the recent Dutch guideline we decided to limit IGFBP-3 determinations in this phase to children below three years of age, because in that age range the 3rd percentile of the reference range of IGF-I is close to the detection limit of most assays (40).

We also advised to add an IGFBP-3 determination when an IGF-I measurement is repeated, for example in children with a low IGF-I but a low pre-test likelihood of GHD. In such children, a low IGFBP-3 (because of its high specificity) considerably increases the likelihood of GHD (or a homozygous *IGFALS* defect). Also if a GH stimulation test is performed, we advise to repeat IGF-I and IGFBP-3 determinations at baseline.

A so far unexplored issue is which cut-off for serum IGF-I should be used for the decision to perform a GH stimulation test. Most papers suggest that IGF-I should be below -2 SDS, but it is obvious that this would imply that approximately 35% of children with GHD would not be detected. In one paper a cut-off of -1 SDS was suggested (49). However, according to general clinical epidemiological principles, the diagnostic value of a test depends on the clinical pre-test likelihood, which implies that different cut-off limits should be used for different ranges of pre-test likelihood.

We therefore calculated the post-test likelihood depending on pre-test likelihood (in five arbitrary categories) and IGF-I result (<-2, <-1 and <0 SDS) (Table 5). Based on this post-test likelihood, the clinician can make the decision if a GH stimulation test would be indicated to confirm or reject the diagnosis of GHD. We appreciate that the decision about which likelihood is sufficiently high to warrant GH stimulation tests is subjective. For the sake of argument we assumed that a post-test likelihood of >10% would be sufficient to decide to perform GH stimulation tests. The consequences of this analysis for different categories are described in the following paragraph. We emphasize that the IGF-I SDS that is used for decision-making should have been adjusted as well as possible for confounding factors such as puberty and nutritional status, although we acknowledge that in contrast to the adjustment for pubertal stage, there are no numerical data that enable the recalculatation of IGF-I SDS adjusted for nutritional status.

For each of the five categories of clinical pre-test likelihood a rational choice of cut-off for serum IGF-I can be made, as explained below. A schematic representation of this advice is shown in Table 6.

**High (≥50%) clinical pre-test likelihood:** In such children a GH stimulation test is indicated regardless of the result of IGF-I. If IGF-I is <0 SDS all post-test likelihoods are high (>52-70%) and even in the rare cases with an IGF-I above 0 SDS [4% in the Dutch study (47)] the post-test likelihood will remain far above 10%. In fact, in children treated for brain tumours, for example using irradiation, or obese children, the IGF-I result is not a determing factor for performing a GH stimulation test (41).

**Moderate (20%) clinical pre-test likelihood:** At a cut-off of 0 SDS the post-test likelihood is approximately equal to the pre-test likelihood, and at a cut-off of -1 SDS it is 30%. We suggest that in such cases a serum IGF-I <0 SDS can be used for the decision to perform a GH stimulation test.

**Rather low (10%) clinical pre-test likelihood:** At a cut-off point of -1.0 SDS the post-test likelihood increases from 10 to 16%, which is in our opinion sufficient to perform a GH stimulation test. When IGF-I is between -1 and 0 SDS the post-test likelihood is not different from the pre-test likelihood and we assume that the clinician’s subjective assessment will influence the decision to perform further testing.

**Low (5%) or very low (≤2%) clinical pre-test likelihood: **At a low pre-test likelihood the post-test likelihood is only above 10% at a cut-off point of -2.0 SDS, while at a very low likelihood the post-test likelihood remains below 10% at all values of the IGF-I results. We suggest that at (very) low pre-test likelihood a rational first step would be to repeat an IGF-I determination, in combination with serum IGFBP-3. If this IGF-I result is in line with the first one and is supported by a low or low-normal IGFBP-3 SDS, further investigations are warranted to diagnose either GHD or rare syndromes characterized by low IGF-I and IGFBP-3. Such syndromes can be divided into two sugroups: those with normal sensitivity to GH [NSD, bio-inactive GH (Kowarski syndrome) or *GHSR* mutation] and those with low or absent sensitivity to GH (GH resistance, such as in children with mutations of *GHR, IGFALS, STAT5B, STAT3* and *IGF1*)(50). In order to differentiate between the two subgroups one can consider performing an IGF-generation test, in spite of its imperfect diagnostic value (51). In a short child with an IGF-I <-2 SDS and GH peak >10 ng/mL one can also consider consulting with a paediatric endocrinologist and/or clinical geneticist to discuss performing a growth-specific whole exome based gene panel (50,52).

Obviously, one should realize that the estimate of the pre-test likelihood of GHD is subjective, and probably will vary between clinicians. The same applies to the cut-off point of post-test likelihood which is considered to be sufficiently high for performing a GH stimulation test.

## Interpretation of a Serum IGF-I >0 SDS in a Short Child

A serum IGF-I >0 SDS can be seen in children with ISS, although mean IGF-I is approximately -1 SDS. If such IGF-I concentrations are found in a short child with low or low-normal birth size or low or low-normal head circumference, this is suggestive of a mutation or deletion of *IGF1R* [for a clinical score, see (53)]. Also, in children with Silver-Russell syndrome (caused by various (epi)genetic disorders, including IGF2 and other gene mutations), a *PAPPA2* mutation, Bloom syndrome and *IGF1* mutations, IGF-I can be increased or in the upper half of the reference range (54).

## Conclusion

In the child referred for short stature and/or growth faltering determination of serum IGF-I is a useful component of laboratory screening. The result should be expressed as SDS for age and sex and adjusted to physiologic (particularly pubertal stage and nutritional status) and analytic factors, and interpreted according to the clinical pre-test likelihood of GHD. The post-test likelihood can guide the clinician’s decision to perform GH stimulation tests.

## Figures and Tables

**Table 1:**
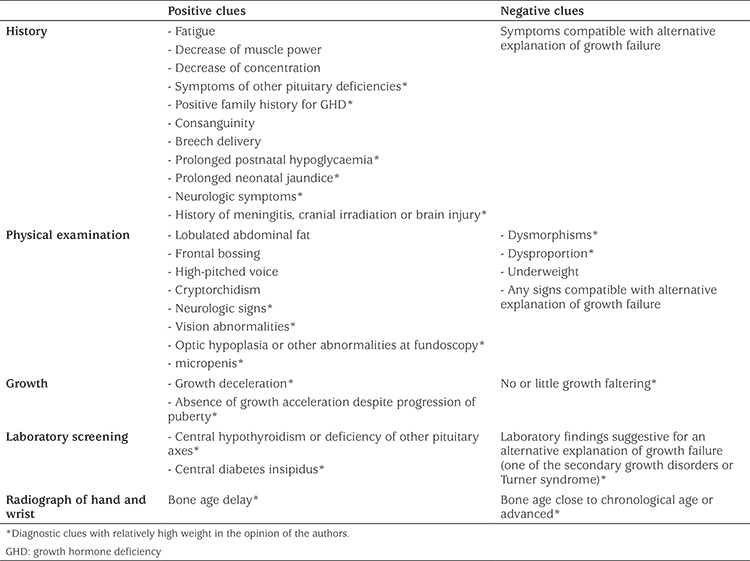
Positive and negative clinical clues for growth hormone deficiency

**Table 2:**
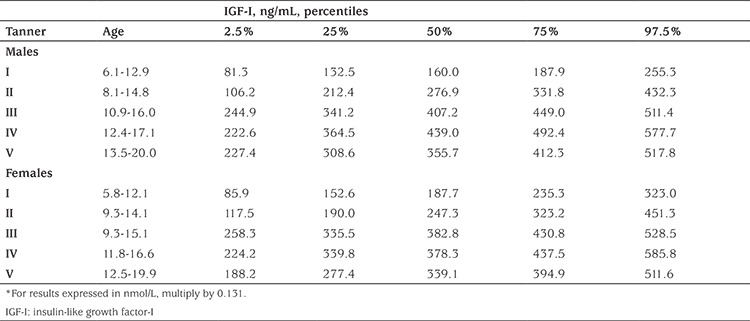
Percentiles of insulin-like growth factor-I (ng/mL) according to sexual maturation, based on 854 samples (age 0-20 years), as measured with iSYS (42)*

**Table 3:**
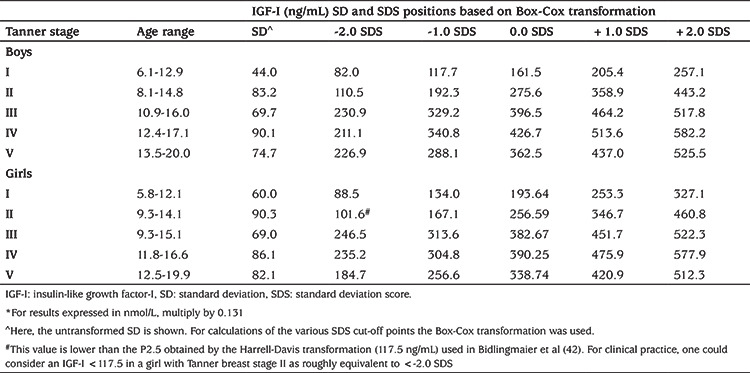
Cut-off points at -2, -1, 0 +1 and +2 SDS of serum insulin-like growth factor-I (ng/mL) according to sexual maturation as measured with iSYS (Bidlingmaier, personal communication 2019)*

**Table 4:**
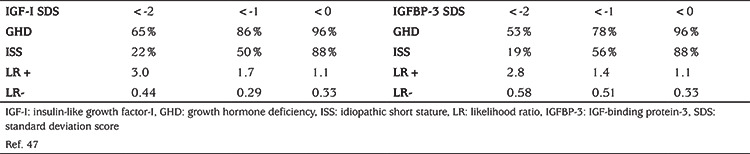
Frequency of children with growth hormone deficiency or idiopathic short stature with a serum insulin-like growth factor-I (IGF-I) or IGF-binding protein-3 <-2, <-1 and <0 standard deviation score, and positive and negative likelihood ratios [likelihood ratio (LR)+ en LR-]

**Table 5:**
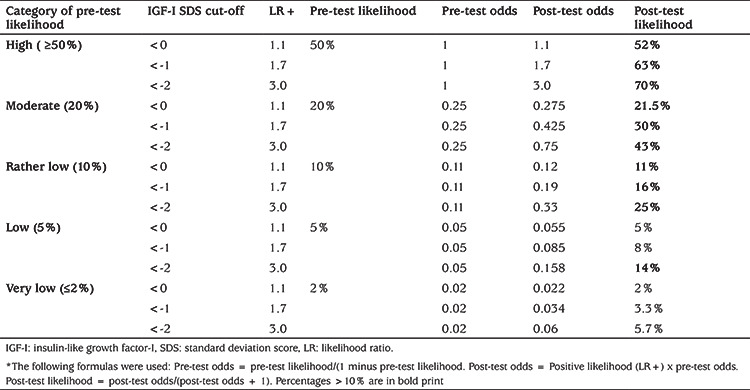
Post-test likelihood of growth hormone deficiency according to clinical pre-testlikelihood and serum insulin-like growth factor-I*

**Table 6:**
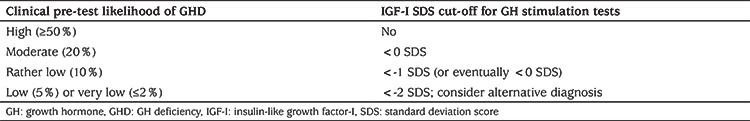
Suggested insulin-like growth factor-I cut-off limits in the screening phase to guide the decision to perform growth hormone stimulation tests
